# The Yin and Yang of bile acid action on tight junctions in a model colonic epithelium

**DOI:** 10.14814/phy2.13294

**Published:** 2017-05-29

**Authors:** Jayashree Sarathy, Sally Jo Detloff, Mei Ao, Nabihah Khan, Sydney French, Hafsa Sirajuddin, Tanushree Nair, Mrinalini C. Rao

**Affiliations:** ^1^ Department of Physiology and Biophysics University of Illinois at Chicago Chicago Illinois; ^2^ Department of Biological Sciences Benedictine University Lisle Illinois

**Keywords:** Chenodeoxycholic acid, epithelial barrier, lithocholic acid, proinflammatory cytokines, reactive oxygen species, tight junctions

## Abstract

Gastrointestinal epithelial barrier loss due to tight junction (TJ) dysfunction and bile acid‐induced diarrhea are common in patients with inflammatory diseases. Although excess colonic bile acids are known to alter mucosal permeability, few studies have compared the effects of specific bile acids on TJ function. We report that the primary bile acid, chenodeoxycholic acid (CDCA), and its 7*α*‐dehydroxylated derivative, lithocholic acid (LCA) have opposite effects on epithelial integrity in human colonic T84 cells. CDCA decreased transepithelial barrier resistance (pore) and increased paracellular 10 kDa dextran permeability (leak), effects that were enhanced by proinflammatory cytokines (PiC [ng/mL]: TNF *α*[10] + IL‐1*ß*[10] + IFN *γ*[30]). CDCA reversed the cation selectivity of the monolayer and decreased intercellular adhesion. In contrast, LCA alone did not alter any of these parameters, but attenuated the effects of CDCA ± PiC on paracellular permeability. CDCA, but not PiC, decreased occludin and not claudin‐2 protein expression; CDCA also decreased occludin localization. LCA ± CDCA had no effects on occludin or claudin expression/localization. While PiC and CDCA increased IL‐8 production, LCA reduced both basal and PiC ± CDCA‐induced IL‐8 production. TNF *α *+ IL1ß increased IFN *γ*, which was enhanced by CDCA and attenuated by LCA. CDCA±PiC increased production of reactive oxygen species (ROS) that was attenuated by LCA. Finally, scavenging ROS attenuated CDCA's leak, but not pore actions, and LCA enhanced this effect. Thus, in T84 cells, CDCA plays a role in the inflammatory response causing barrier dysfunction, while LCA restores barrier integrity. Understanding the interplay of LCA, CDCA, and PiC could lead to innovative therapeutic strategies for inflammatory and diarrheal diseases.

## Introduction

Bile acids synthesized from cholesterol in the liver are secreted into the intestine where they aid in fat digestion and can also serve as signaling molecules (Alrefai and Gill 2007; Hofmann 2009). Conjugation to taurine or glycine in the liver increases bile acid solubility. The primary bile acids, chenodeoxycholic acid (CDCA) and cholic acid (CA), are formed in the liver, and in the intestine they sequentially undergo deconjugation and dehydroxylation by bacterial hydrolases. The latter step results in the formation of secondary bile acids, deoxycholic acid (DCA) from CA and lithocholic acid (LCA) from CDCA, with the bulk of LCA formation occurring in the colon. Over 95% of the intestinal luminal bile acids are reabsorbed in the distal ileum and recycled back to the liver via enterohepatic circulation. In normal physiology, the bile acid pool size, defined as the total amount of bile acids in the enterohepatic circulation is as follows in mg: Total: 1300–3650; CA: 500–1500; CDCA: 500–1200; DCA: 250–800; and LCA: 50–150 (Dawson 2016). While the 5% of bile acids (*μ*mol/L range) entering the colon under normal conditions modulate cell proliferation, gut‐associated immunity, motility, and ion transport (Alrefai and Gill 2007; Hofmann 2009), it does not increase fluid secretion.

In contrast, in pathophysiological conditions such as irritable bowel syndrome (IBS), inflammatory bowel disease, and ileal resection, bile acid recycling is compromised resulting in high(mmol/L) concentrations of bile acids in the colon (Martinez‐Augustin and Sanchez Medina 2008), and increase in CA and CDCA in human fecal samples (Bajor et al. 2010). This excess of bile acids (>3 mmol/L) in the colon impairs many functions, resulting in genomic instability and cancer, epithelial barrier disruption, and increased fluid accumulation resulting in diarrhea (Dharmsathaphorn et al. 1989; Hofmann 1999; Robb and Matthews 2005; Halpern et al. 2006; Jean‐Louis et al. 2006; Raimondi et al. 2008; Bernstein et al. 2011; Islam and DiBaise 2012; Ao et al. 2013). A major consequence of diarrheal diseases is fluid loss and subsequent dehydration, therefore it is important to understand the mechanisms underlying bile acid‐associated diarrhea. For example, of the 90 million patients worldwide annually suffering from IBS, at least 10 million suffer from diarrhea (IBS‐D) induced by bile acid malabsorption (Islam and DiBaise 2012). In IBS‐D patients, fecal content of primary bile acids were significantly higher (15.9 ± 5.6%) compared to healthy subjects (4.1 ± 0.4%), whereas LCA showed no increase (Duboc et al. 2012). Although excess colonic bile acids are known to alter mucosal permeability and fluid secretion, few studies have compared the effects of specific bile acids known to alter Cl^‐^ secretion on tight junction function.

Not surprisingly, the various bile acids differ in the way they alter intestinal function (Martinez‐Augustin and Sanchez Medina 2008). Thus, the 7*α*‐dihydroxy bile acids, CDCA and DCA, are prosecretory in the colon (Mekjian et al. 1971; Chadwick et al. 1979; Mekhjian et al. 1979; Martinez‐Augustin and Sanchez Medina 2008; Bajor et al. 2010; Ao et al. 2013; Domingue et al. 2016), acting largely from the basolateral surface of colonic epithelia, both in cell lines and in ex vivo rabbit and human preparations. This raises the yet unanswered physiological question of how bile acids entering the colon in the lumen, cross the epithelial barrier to reach the basolateral side (Ao et al. 2013). In contrast, CA (Martinez‐Augustin and Sanchez Medina 2008; Ao et al. 2013) and the 7*α* enantiomer of CDCA, ursodeoxycholic acid (UDCA), do not directly alter intestinal ion secretion (Martinez‐Augustin and Sanchez Medina 2008). In the human colon carcinoma cell line, T84, a model of a secretory epithelium, UDCA attenuated cAMP and Ca^2+^‐dependent Cl^−^ secretion (Kelly et al. 2013) and CA had no effects. We and others have shown that LCA does not activate Cl^−^ secretion, but attenuates the prosecretory effects of cAMP‐dependent agents in T84 cells (Ao et al. 2016).

Epithelial integrity is critical for the intestinal mucosa to function as a barrier. Agents, including bile acids, that alter the epithelial architecture and/or the paracellular pathway, modify barrier function and when disruptive can lead to dysfunction. The intestinal barrier is established by the interplay of distinct morphological structures, especially the tight junction which serve both as a gate to influence paracellular transport and as a fence to maintain epithelial cell polarity. As a gate, tight junctions act both as a pore pathway to allow ion movement, measured as transepithelial resistance (TER), and as a leak pathway, measured as permeability to larger molecules, to allow macromolecular transit. Bile acids are reported to regulate tight junctions and their actions depend on their type, concentration, cell type studied, and side of exposure to the epithelial layer (Araki et al. 2005; Munch et al. 2007; Catalioto et al. 2008; Hughes et al. 2008; Raimondi et al. 2008). In human colonic biopsies, CDCA (1 mmol/L) or DCA (0.5–1 mmol/L) decreased TER, increased Cr‐EDTA permeability and increased *E. coli* uptake (Munch et al. 2007); furthermore, lower concentrations (100 *μ*mol/L) of DCA or CDCA increased translocation of *E. coli*, and therefore permeability, without altering TER (Munch et al. 2011).

Barrier function in response to various bile acids (50–250 *μ*mol/L) has been studied extensively in the human colon carcinoma cell line, Caco‐2, which is more representative of an absorptive epithelium. For example, Araki et al. (2005) reported that at 200 *μ*mol/L CA (and its glycol‐ and tauro‐ conjugates), DCA, CDCA, and UDCA decreased TER. In contrast, Raimondi et al. showed that at 50 *μ*mol/L, while CA, DCA, and CDCA increased dextran flux, UDCA had no effect (Raimondi et al. 2008). Furthermore, Hughes et al. (Hughes et al. 2008) reported that only DCA and LCA, but not CDCA, decrease TER and increase ^14^C‐mannitol flux in these cells. Finally, a mixture of CA, DCA, and taurocholic acid (0.5–4.5 mmol/L), increased paracellular transport of otilionium bromide, a drug used to treat IBS, without altering transcellular transport in Caco‐2 cells (Catalioto et al. 2008). While bile acids are known to alter some ion transport processes in Caco‐2 cells, these effects have not been correlated with changes in paracellular permeability (Alrefai et al. 2007).

Tight junctions are comprised of many proteins including occludins, claudins, JAM‐1, and ZO‐1 and reports on the effects of bile acids on their distribution are varied. Thus, dietary fat and bile acids (1.5–3 mmol/L; ≈ 10–20% of that in rat bile) increase permeability, and decrease expression of claudin‐1, ‐3 and JAM‐1 in Caco‐2 monolayers (Suzuki and Hara 2010). In contrast, in these cells, Raimondi et al. (2008) showed a rearrangement/dephosphorylation of occludin, accompanying bile acid‐induced permeability changes, but Hughes et al. (2008) reported no change in occludin.

While many studies probing bile acid action examine the effects of individual bile acids, it is important to also study them in combination to provide insights into in vivo conditions. A case in point are studies on the effects of bile acids on cytotoxicity (Barrasa et al. 2013) and apoptosis. In addition to being cell‐ and bile acid‐specific (Araki et al. 2003), the cytotoxicity of a particular bile acid can be ameliorated by the presence of other bile acids. Thus, the cytotoxicity of hydrophobic DCA is attenuated by the more hydrophilic bile acids CA, hyocholic acid and UDCA, or by CDCA in the presence of UDCA and CA in the rat intestinal IEC‐6 cells, and in Caco‐2 cells (Araki et al. 2003).

Overall, there have been few studies comparing the effects of different bile acids known to affect ion transport, added alone and in combination on epithelial paracellular permeability. With the caveat that a comprehensive examination of all bile acids on epithelial permeability is beyond the scope of any single study, of the four major bile acids, we examined whether CDCA and its dehydroxylated product, LCA, affect barrier integrity in T84 cells based on the following rationale. Of the two primary bile acids, we have shown in T84 cells that while CA has no effect (J. Domingue, M. Ao, J. Sarathy, and M. C. Rao, unpubl. observations), CDCA stimulates Cl^−^ secretion (Ao et al. 2013; Domingue et al. 2016). Of the two major secondary bile acids, DCA has effects similar to CDCA (Binder and Rawlins 1973; Dharmsathaphorn et al. 1989; Ao et al. 2013), whereas LCA is antisecretory at the concentrations (0.005–0.05 mmol/L) found in the colon (Ao et al. 2016). Of the two prosecretory bile acids, CDCA is more potent and present in larger amounts in normal physiology (Dawson 2016).

Short‐term (<1 h) exposure to 500 *μ*mol/L CDCA‐stimulated Cl^−^ secretion via activation of the cystic fibrosis transmembrane conductance regulator (CFTR) and involved both intracellular cAMP and Ca^2+^ signaling (Ao et al. 2013; Domingue et al. 2016). While acute exposure to LCA (5–500 *μ*mol/L) does not alter secretion, it attenuates cAMP‐dependent, but not Ca^2+^‐mediated Cl^−^ secretion even at 50 *μ*mol/L. In short‐term (≈30 min) Ussing chamber studies, neither CDCA nor LCA altered TER (Ao et al. 2016). However, Kelly et al. (2013) reported that at longer time points (>1 h) DCA decreased TER in T84 cells.

Therefore, in this study, we examined if longer exposure to CDCA and LCA, at concentrations known to alter Cl^−^ secretion, would affect T84 monolayer permeability. We studied CDCA and LCA effects on pore and leak functions (i.e., gate properties), at the maximal concentrations at which they modified Cl^−^ secretion. These amounts are below their critical micellar concentration (CMC: >2 mmol/L for CDCA (Hofmann and Mysels 1992); >0.9 mmol/L for LCA (Hofmann and Roda 1984)). In different models, inflammatory mediators, ROS, and bile acids have been linked to alterations in function, but the direct interplay of the three cascades and a specific function, such as epithelial permeability, has not been examined. Therefore, we also examined the role of inflammatory mediators and ROS in CDCA and LCA action. Using a scavenger of ROS, we demonstrate that ROS plays an important role in CDCA‐induced barrier dysfunction. Our data demonstrate for the first time that CDCA and its dehydroxylated derivative, LCA, have distinct, and at times opposite effects on epithelial barrier function. CDCA's role is akin to that of some proinflammatory cytokines, involves ROS, and affects both pore and leak functions, whereas LCA ameliorates the leak actions of CDCA.

## Material and Methods

### Materials

T84 human colon carcinoma cells were obtained from American Type Culture Collection (Manassas, VA). Transwell tissue culture inserts, (6.5‐mm, 0.4‐ *μ*mol/L pore size; Corning 3413) were purchased from Corning Inc. Life Sciences (Lowell, MA). Dulbecco's Modified Eagle Medium (DMEM), Ham's F‐12 nutrient mixture, and bovine calf serum were obtained from Invitrogen (Carlsbad, CA). LCA, CDCA, etoposide, N‐acetyl cysteine (NAC), protease inhibitor cocktail, and phosphatase inhibitor cocktail 2 were purchased from Sigma‐Aldrich Corp. (St. Louis, MO), 10 kDa cascade blue dextran was obtained from Life Technologies, (Grand Island, NY), and Dispase II, and antibodies to occludin and claudin were purchased from Thermofisher Scientific (Waltham, MA). The following kits were purchased: Lactate Dehydrogenase (LDH) Cytotoxicity Assay Kit and Apoptosis Detection Kit 1 from Pierce Thermofisher Scientific; IL‐8, IFN*γ*, and TNF*α* ELISA kits from BD Biosciences (San Jose, CA); hydrogen peroxide colorimetric detection kit from Enzo Life Sciences Inc (Farmingdale, NY). Unless otherwise specified, all other reagents were of analytical grade, and were purchased from either Sigma‐Aldrich Corp. or Fisher Scientific (Hanover Park, IL).

### Cell Culture

T84 cells were grown to confluency in commercially available, “tissue culture‐treated” 6‐well, 24‐well, or 96‐well plates, and collagen‐coated 24‐well Transwells in DMEM/F‐12 medium containing bovine calf serum, penicillin (100 U/mL), streptomycin (100 *μ*g/mL), and ampicillin (8 *μ*g/mL) as described earlier (Ao et al. 2011). Since the support on which cells are grown (i.e., solid or permeable) are known, at times, to influence phenotype, in a few experiments (e.g., Fig.  9) we have confirmed the findings in both six‐well plates and collagen‐coated Transwells. In all experiments we have demarcated the type of support used. We have reported that these cells were female as determined by RT‐PCR amplification of the amelogenin gene (Ao et al. 2016). Cells, between passages 39 and 50, were incubated in a humidified atmosphere of 5% CO_2_ at 37°C and grown in Transwells until transepithelial resistance (TER) reached 1000 Ω cm^2^ (generally 2–3 weeks). They were then used to measure bile acids effect on TER, paracellular permeability, dilution potentials, and protein expression. For all time course experiments, cultures were treated with the various agents in serum‐free medium. While serum is needed for the cells to reach confluence, we (Ao et al. 2016; Domingue et al. 2016) and others (Kelly et al. 2013) have found that studying signaling cascades in T84 cells in the presence of serum, often leads to inconsistencies in data, due to the undefined and variable composition of serum. Therefore, to provide reproducible assay conditions, we use a serum‐free regimen for up to 24 h.

### Cell viability and apoptosis assays

Cellular viability and apoptosis were studied by flow cytometry (BD Accuri Flow cytometer, San Jose, CA) following staining with Propidium Iodine (PI) and FITC‐labeled Annexin V (Apoptosis Detection Kit I), respectively. T84 cells were grown to confluency on six‐well plates and incubated at 37°C overnight (O/N; generally ≥ 18 h), with 500 *μ*mol/L CDCA and 50 *μ*mol/L LCA, alone or in combination and ± PiC; etoposide (10 *μ*mol/L, 1 h or O/N) was used as a positive control for apoptosis. After exposure to agents, cells were labeled for 15 min in the dark, with FITC‐Annexin V at room temperature (RT) and PI as a standard flow cytometry viability probe, as per manufacturer's instructions.

Cytotoxicity effects were measured using the LDH Kit as previously reported (Ao et al. 2016). Briefly, T84 cells (~4000 cells per well) were grown in 96‐well plates O/N and treated with DMSO (0.2%) or different concentrations of bile acids for varying time points. The LDH release was measured as absorbance at 490 nm and background signal at 680 nm, as per manufacturer's protocol. Maximal LDH release was measured after cells were treated with 10× lysis buffer and results are reported as percent cytotoxicity.

### Intercellular adhesion assay

T84 cells were grown in 12‐well plates until 100% confluent. Monolayers were treated with DMSO (0.2%), CDCA (500 *μ*mol/L), or LCA (50 *μ*mol/L) in Hanks’ balanced salt saline with calcium [2.1 mmol/L] and magnesium [1.41 mmol/L] (HBSS‐CM) for different times in the tissue culture incubator (5% CO_2_, 37°C). After treatment, the monolayers were washed once with HBSS‐CM and incubated with dispase solution (20 mg/mL in HBSS‐CM) for 30 min in cell culture incubator. After the incubation, the plate was placed on a shaker for 5 min. Pictures were then taken against a black background to document the integrity of the monolayer (Jiang et al. 2014).

### Barrier function studies

#### Transepithelial resistance

For barrier function studies, T84 cells were seeded at a density of ~250,000 cells per insert into 6.5‐mm, 0.4‐*μ*mol/L pore size collagen‐coated Transwells and grown until TER reached 1000 Ω cm^2^. TER was measured with an EVOM voltohmmeter and chopstick electrodes (World Precision Instruments, Sarasota, FL). The monolayers were serum‐starved overnight in the presence or absence of bilateral addition of a combination of cytokines (PiC [ng/mL]: TNF*α* [10] + IL‐1ß [10] + IFN*γ*[30]) and TER was measured. These concentrations of PiC were used in all subsequent experiments. The effects of apical exposure to CDCA (500 *μ*mol/L), LCA (50 *μ*mol/L), or CDCA +LCA on TER were studied at various time points, as indicated, over a 20 h period.

#### Dilution potential

The pore function was studied by measuring dilution potentials (DΨ) across the tight junctions in response to a unilateral 50% reduction in NaCl concentration (Rao 1998). T84 cells grown to confluency in transwells were exposed to Buffer A, in the basolateral chamber, consisting of (in mmol/L): 120 NaCl, 10 HEPES, pH 7.4, 5 KCl, 10 NaHCO_3_, 1.2 CaCl_2_, and 1 MgSO_4._ The transmonolayer potential, Ψ _[A‐Buffer]_, was measured in mV, using EVOM voltohmmeter and chopstick electrodes, with the reference electrode in the basolateral medium (BLM) and recording electrode in the apical medium (AM). The buffer A in the apical chamber was replaced with 0.5 mL of buffer B, containing in mmol/L: 60 NaCl, 120 mannitol, 10 HEPES, pH 7.4, 5 KCl, 10 NaHCO_3_, 1.2 CaCl_2_, and 1 MgSO_4_ and the potential *Ψ*_(B‐buffer)_ recorded in mV over 30 mins, until it reached a steady value. These measurements were repeated in wells treated with CDCA (500 *μ*mol/L; AM), or LCA (50 *μ*mol/L; AM), or a combination of CDCA and LCA (AM) ± NAC (1 mmol/L). The potential across a blank filter in buffer A was also measured and subtracted from the *Ψ* values obtained from monolayers grown on the filter membrane insert. The D*Ψ* was calculated using the formula Δ*Ψ* = (*Ψ*_[A‐Buffer]_ –*Ψ*_[Blank]_)–*Ψ*_[B‐buffer]_) (Buchert et al. 2012).

#### Dextran flux

Cells were grown in Transwells as described for TER measurements, serum‐starved O/N ± bilateral exposure to PiC ±  bilateral exposure to NAC [1 mmol/L], followed by treatment with CDCA (500 *μ*mol/L, AM or BLM), or LCA (50 *μ*mol/L; AM), or CDCA(AM)+LCA(AM). The tight junction function was measured in these cells by following the movement of cascade blue‐labeled 10‐kDa dextran from the AM to the BLM over time. Net dextran flux was calculated by subtracting the dextran adhered to the membrane of blank, unseeded Transwells. In the published literature, dextran movement has been reported generally as the amount transferred (Rao 1998) and less frequently as flux units (amount/time)(Buchert et al. 2012). Therefore, we decided to show some of our data using the former representation and others using the latter. Flux is reported as the amount of dextran that moved across the monolayer over the time period noted in each panel, in *μ*g/mL (Fig.  3) or as apparent permeability P_app_ = (dQ/dt)/A*C; where dQ is the concentration of dextran in BLM at time interval *t*, A is the cross‐sectional area of the Transwell membrane and C is the starting concentration of dextran in AM ( Fig. 4, (Buchert et al. 2012)).

### Cytokine assays

All cytokine assays were performed using culture supernatants of serum‐starved cells treated with bile acids. Cells were grown to confluency in 24‐well plates, serum‐starved, and exposed to CDCA (500 *μ*mol/L) and/or LCA (50 *μ*mol/L) and/or PiC for different times. To determine if growing cells on permeable supports would affect the cytokine response, T84 cells were also grown on transwells and exposed to AM CDCA (500 *μ*mol/L) and/or AM LCA (50 *μ*mol/L) and/or bilateral PiC for different times. The supernatants were collected and stored at −80°C. Cytokines in the samples were measured using a sandwich ELISA with appropriate antibodies as detailed below. TNF*α* was measured using anti‐human TNF*α* monoclonal antibody in supernatants of cells treated with increasing doses of CDCA and LCA for 1, 2, and 18 h. IL‐8 was measured using anti‐human IL‐8 monoclonal antibody in samples from cells treated O/N with CDCA (500 *μ*mol/L), LCA (50 *μ*mol/L), a combination of CDCA (500 *μ*mol/L) and LCA (50 *μ*mol/L) ± PiC. TNF*α* (100 ng/ml) was used as positive control. IFN*γ* was assayed using anti‐human IFN*γ* monoclonal antibody in supernatants collected from cells treated with CDCA (500 *μ*mol/L), LCA (50 *μ*mol/L), or CDCA (500 *μ*mol/L) + LCA (50 *μ*mol/L) ± (TNF*α*+IL‐1ß;10 ng/mL each) for varying time periods. The ELISAs were performed using the manufacturer's protocol. In brief, 96 wells were coated with the monoclonal antibody against the specific cytokine, standards and samples incubated, washed and treated with streptavidin‐horseradish peroxidase conjugate mixed with a biotinylated anticytokine antibody. The resulting antibody‐antigen‐antibody “sandwich” was detected using a TMB substrate. The reaction was stopped with phosphoric acid, and absorbances in each microwell were read at 450 nm within 30 min with a *λ* correction measured at 570 nm. Cytokine levels were quantified against recombinant human cytokine standards provided in the kit. Each dataset represented a separate batch of cells; in all but one experimental setup, the samples were run in triplicate in each set and averaged as *n* = 1; in the data on PiC + CDCA + LCA shown in Figure 9B, the samples were run in duplicates.

### Reactive oxygen species assays

To examine the effects of bile acids on reactive oxygen species (ROS), two approaches were used. T84 cells (~20,000 cells) were grown in 24‐well plates, serum‐starved and treated O/N with CDCA (500 *μ*mol/L), or LCA (50 *μ*mol/L), or CDCA (500 *μ*mol/L) + LCA (50 *μ*mol/L) ± PiC ± NAC. Nuclear and mitochondrial ROS production was assessed by staining with Cell Rox Green and flow cytometry. This method, however, does not account for ROS released from the cells. Therefore, the metabolic by‐product of ROS, hydrogen peroxide (H_2_O_2_), was measured in cell culture supernatants of T84 cells grown in 24‐well plates, serum‐starved and treated with CDCA (500 *μ*mol/L), or LCA (50 *μ*mol/L), or CDCA (500 *μ*mol/L *μ*mol/L) + LCA (50 *μ*mol/L) ± PiC for 4 h, or O/N treatment. H_2_O_2_ was detected using a colorimetric detection kit as per manufacturer's protocol. Briefly, buffer blanks, standards and samples were assayed in wells in the presence of a dye, xylenol orange, incubated for 30 min; the absorbances were read at 550 nm and the mean optical density of the blank wells subtracted from each well. The reaction produces a purple color proportional to the concentration of H_2_0_2_ in the sample. To validate the ROS assay, in limited experiments, murine RAW 267.4 macrophages, a known model of ROS generation, was studied. Cell culture supernatants from ~ 20,000 RAW 267.4 macrophages treated with PiC, CDCA (500 *μ*mol/L), or LCA (50 *μ*mol/L) were assayed to study cell specificity of bile acid action.

### Western blotting and immunodetection

T84 cells grown in 12‐well plates were serum‐starved O/N ± PiC followed by AM treatment of CDCA (500 *μ*mol/L), LCA (50 *μ*mol/L), or a combination of CDCA and LCA. Cells were then lysed in a buffer containing in mmol/L: 1 EDTA, 2 MgCl_2_, 5 *β*‐mercaptoethanol, 1 DTT, 25 Tris·HCl, pH 7.4, and protease inhibitor cocktail. Cell membranes were isolated by centrifugation of the total cell lysate for 30 min at 100,000*g*. SDS‐PAGE and western blotting were performed as described earlier (Ao et al. 2011). Briefly, 30 *μ*g protein from total lysate were loaded on a gradient polyacrylamide gel, transferred to a PVDF membrane (Millipore, Billerica, MA), blocked with 5% BSA in Tris‐buffered saline containing 0.1% Tween‐20 (TBST), and were then incubated with occludin‐ or claudin‐polyclonal antibodies (1 *μ*g/mL) in 1% BSA in TBST O/N at 4°C. The membranes were then washed with TBST, and incubated with horseradish peroxidase‐conjugated secondary antibody for 1 h at RT. The antigen‐antibody complex was visualized using SuperSignal West Pico/Femto chemiluminescent substrate kit (Thermo Fisher Scientific, Waltham, MA). The blots were stripped and reprobed with GAPDH mAb (1:2000) to normalize for protein loading.

### Immunolocalization

T84 cells were grown on 24‐Transwell inserts until the TER reached ~1000 Ω cm^2^. Monolayers were treated apically with DMSO (0.2%), CDCA (500 *μ*mol/L), and LCA (50 *μ*mol/L) alone or in combination for different time periods. The cells were washed once with wash buffer (PBS + 1 mmol/L CaCl_2_) and fixed with 4% formaldehyde for 20 min. Cells were washed 3 × 5 min with wash buffer, and then blocked with 5% normal goat serum in wash buffer containing saponin (0.8 g/1 L) for 1 h at RT. After blocking, cells were incubated with Alexa Fluor^®^ 594‐conjugated occludin antibodies (1:100, mouse monoclonal) for 1 h at RT. The cells were then washed three times with wash buffer containing saponin and mounted on slides using SlowFade^®^ Gold Antifade Mountant from Molecular Probes (Waltham, MA). The images were captured using a Zeiss LSM 710 confocal microscope (META) (Zeiss, Oberkochen, Germany). Images were taken using diode UV laser, Ex_*λ*_ 405 nm, Em_*λ*_ 420–480 nm, and DPSS 561 laser, Ex_*λ*_ 561 nm, Em_*λ*_ 600–650 nm. A 63x/1.46 oil objective was used, and the final magnification shown is 126X.

### Statistical analysis

All experiments were performed at least three times each (*n* ≥ 3). For kit assays, samples were run in triplicates and as mentioned in duplicates in one instance, and the values averaged to obtain *n* = 1. Data are presented as mean ± SEM. One‐way ANOVA followed by Tukey's or Dunnet's test was performed to test statistical significance. A *P* < 0.05 was considered to be statistically significant.

## Results

### Paracellular Permeability Studies

**Figure 1:**
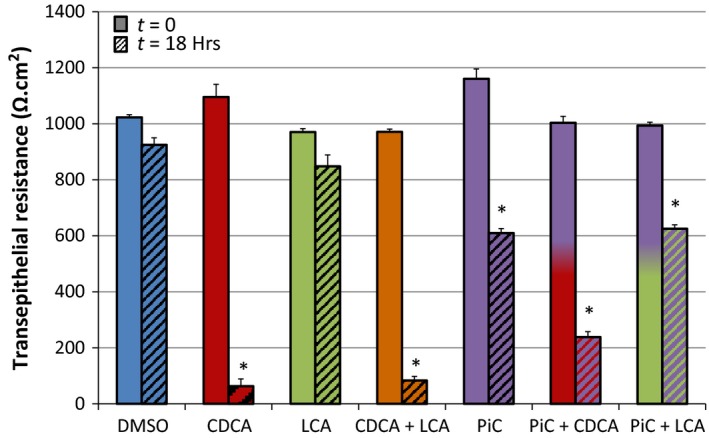
Effects of apical exposure to CDCA and LCA on transepithelial resistance (TER) changes in the presence or absence of PiC. T84 cells were grown on collagen‐coated Transwell inserts until the TER reached 1000 Ω cm^2^. They were then apically treated O/N with DMSO (0.2%), CDCA (500 *μ*mol/L), LCA (50 *μ*mol/L), or CDCA + LCA, O/N PiC ([ng/mL]: TNF *α* [10] + IL‐1*ß* [10] + IFN *γ*[30]), PiC + CDCA, or PiC + LCA. TER was measured before and after treatments using chopstick electrodes. *P < 0.05 (*n* = 3) as compared with control at *t* = 0.

The actions of bile acids on the gate mechanisms of paracellular permeability were studied in T84 cells grown on Transwells. We first examined their effects on TER as a measure of epithelial barrier function. Since we (Ao et al. 2013, 2016; Domingue et al. 2016) and others had reported that short‐term (<30 min) bile acid treatment had no effect on TER, we first focused on examining the effect of long‐term (≥18 h) CDCA and LCA on TER. Using the maximal effective concentrations used in our earlier studies (Ao et al. 2013, 2016; Domingue et al. 2016), overnight treatment of T84 cells grown on Transwells, with CDCA (500 *μ*mol/L) caused a 95% decrease in TER (AM 18 h (O/N) treatment: Control: 1096 ± 45; 500 *μ*mol/L CDCA: 63 ± 2 Ω cm^2^ *n* = 4; Fig. 1), and this decrease was seen only when AM was exposed to CDCA (O/N BLM treatment, 18 h: 1039 ± 29 Ω cm^2^). Hence, in all future experiments, AM of cells were exposed to bile acids. LCA alone (AM or BLM) did not affect TER (18 h: Control: 925 ± 15; 50 *μ*mol/L LCA: AM 848 ± 41 Ω cm^2^ (*n* = 6); BLM: 879 ± 55 Ω cm^2^ (*n* = 3)) (Fig. 1). Further, in limited experiments, we also found that overnight treatment with either lower or higher doses of LCA did not have an effect (TER, Ω.cm^2^: Control: 924.8 + 14 (*n* = 3); LCA 5 *μ*mol/L: 915.2 (*n* = 2); LCA 500 *μ*mol/L: 901 + 31 (*n* = 3)). Overnight exposure to a cocktail of proinflammatory cytokines (PiC [ng/mL]: TNF*α* [10] + IL‐1*ß* [10] + IFN*γ*[30]) caused an expected decrease in TER (Fig. 1). This TER decrease was further accentuated by an additional (1 h) exposure to CDCA, but not to LCA.

**Figure 2:**
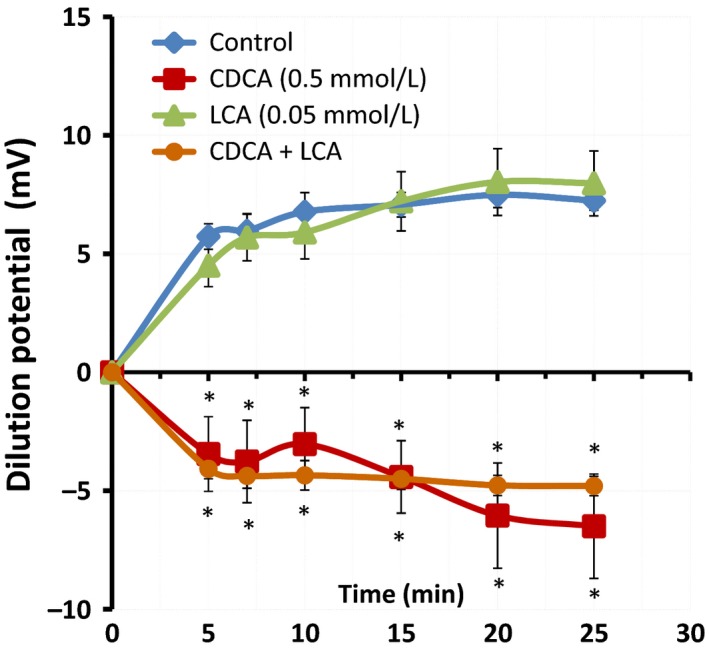
Effects of CDCA and LCA on ion selectivity. Cells grown on collagen‐coated Transwells inserts were used to measure dilution potentials (D*ψ*). Potential *ψ*_[A‐Buffer]_ and *ψ*_[B‐Buffer]_ were measured in mV as described in Materials and Methods. Briefly, the initial *ψ* was recorded with Buffer A (containing 120 mmol/L NaCl) in the apical chamber, using chopstick electrodes and EVOM voltohmmeter. Then the Buffer A was replaced with Buffer B (containing 60 mmol/L NaCl and 120 mmol/L mannitol) and *ψ* was recorded until it reached a steady level (25 mins). Buffer B contained DMSO (control), CDCA (500 *μ*mol/L), LCA (50 *μ*mol/L), or CDCA + LCA. *P < 0.05 as compared with control, *n* = 4 for CDCA + LCA, and *n* ≥ 9 for other groups.

Since the drop in TER indicates an increase in the electrical conductance of the barrier and an alteration in the pore function, we examined if it is accompanied by a change in junctional charge selectivity. Dilution potentials (D*Ψ*), in response to bile acid exposure, were measured at 5 min intervals over 30 min, after a 50% dilution of mucosal buffer. As seen in Figure 2, the control T84 cells exhibited a cation‐selective paracellular barrier with a D*Ψ* of 7.5 + 0.5 mV. While LCA did not alter DΨ (8.0 + 1.3 mV; *n* = 7), CDCA dramatically reversed the D*Ψ* to −6.5 + 2.2 mV (*n* = 7), suggesting altered pore charge selectivity. Similar to our findings in TER, in combination with CDCA, LCA was unable to restore D*Ψ* to control values.

**Figure 3:**
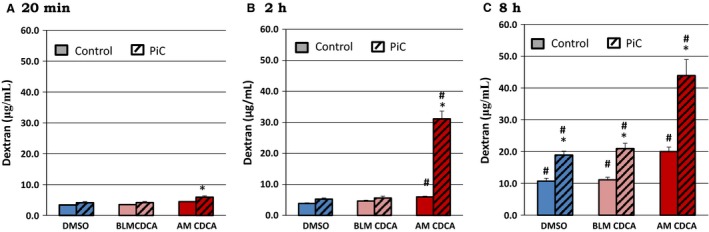
Effects of CDCA (500 *μ*mol/L; Apical media (AM) or basolateral media (BLM)) on paracellular permeability as measured by Cascade Blue^®^‐labeled 10 kDa dextran flux. Cells were grown on collagen‐coated Transwell inserts until the TER reached 1000 Ω cm^2^. Cells were serum‐starved overnight in the presence or absence of PiC followed by DMSO (control), CDCA (AM or BLM) for 20 min, 2 h, or 8 h. The movement of Cascade Blue^®^ ‐labeled 10 kDa dextran from the AM to the BLM was measured over time. *P < 0.05 comparing control±CDCA with PiC ± CDCA; ^#^ *P* < 0.05 comparing over time; *n* = 4.

**Figure 4:**
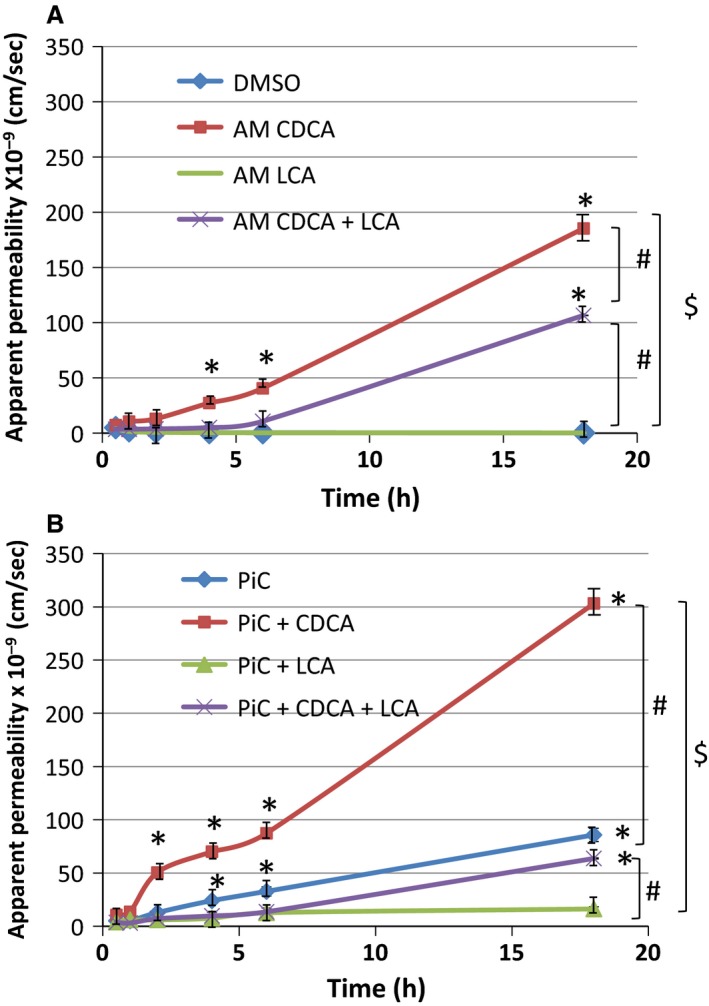
Effects of CDCA (500 *μ*mol/L), LCA (50 *μ*mol/L) and/or PiC on paracellular permeability as measured by Cascade Blue^®^‐labeled 10 kDa dextran flux. Dextran flux was measured as described in Figure 3. Cells were serum‐starved overnight in the absence (A) or presence (B) of PiC followed by apical exposure (AM) to DMSO (control), CDCA, or LCA, or a combination of CDCA and LCA. Dextran flux was expressed as apparent permeability (cm/sec), which was calculated as described in the Materials and Methods. *P < 0.05 compared with control; *n* = 4.

We next studied the leak pathway of the tight junction by examining the effect of bile acids on the movement from AM → BLM of 10 kDa Cascade Blue^®^ dextran, a probe often used to study paracellular permeability (Raimondi et al. 2008). The molecular radius of 10 kDa dextran is ~23.6 Å (Al‐Sadi and Ma 2007) and is similar to the molecular radii (10–25 Å) of bile acids (Whiting 1986). As shown in Figures 3 (depicted as amount of dextran) and 4A, (depicted as apparent permeability) 500 *μ*mol/L CDCA increased 10 kDa dextran fluxes over time (8–18 h), only from the apical side (Fig. 3) and the increases in basal and CDCA‐induced permeability were enhanced in the presence of PiC (Fig. 4B). Neither the effects of AM CDCA at 20 min and 2 h, nor the effects of BLM CDCA at 20 min, 2, or 8 h were different from DMSO. In contrast, 50 *μ*mol/L LCA significantly attenuated increases in dextran fluxes induced by CDCA alone, PiC alone, or CDCA + PiC in combination (Fig. 4A and B) (18 h, in *μ*g: Control: 7 ± 1; LCA: 5 ± 1; PiC: 51 ± 4; PiC + LCA: 10 ± 1; CDCA: 110 ± 2; PiC+CDCA: 180 ± 2; CDCA+LCA: 63 ± 1; PiC+CDCA+LCA: 37.8 ± 0.3; *n* ≥ 4). In Figure 4, we report dextran flux as apparent permeability, (P_app__), cm/sec to account for surface area and initial concentration of dextran in the apical compartment. The P_app__ clearly increases with time and CDCA or PiC treatment. Also evident is the additive effect of PiC + CDCA, seen as early as 2.5 h and continuing to increase up to 18 h. Interestingly, the ability of LCA to attenuate CDCA‐induced permeability decreases over time; for example, it is 81% and 74% at 4 and 6 h, respectively, but only 43% at 18 h. In contrast, LCA inhibits the PiC and PiC+CDCA effect more robustly.

**Figure 5:**
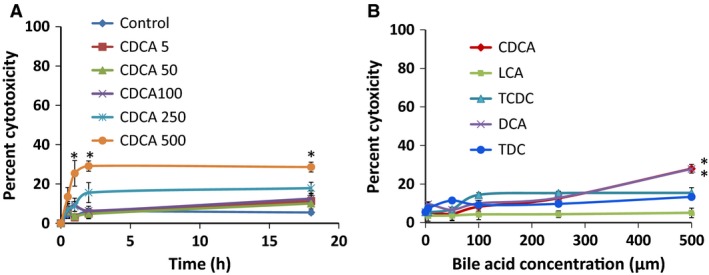
Effects of bile acids on cytotoxicity as measured by lactate dehydrogenase (LDH) release. Cytotoxicity was measured using the LDH assay Kit as described in Materials and Methods. Briefly, T84 cells grown in 96‐well plates were apically treated with DMSO (control), increasing doses of CDCA (5–500* μ*mol/L) for 0.5 h, 1 h, 2 h, or overnight (A) and increasing doses of different bile acid for 18 h (B) and LDH release was measured spectrophotometrically at 490 nm. *P < 0.05, compared with DMSO treatment; *n* = 4.

**Figure 6:**
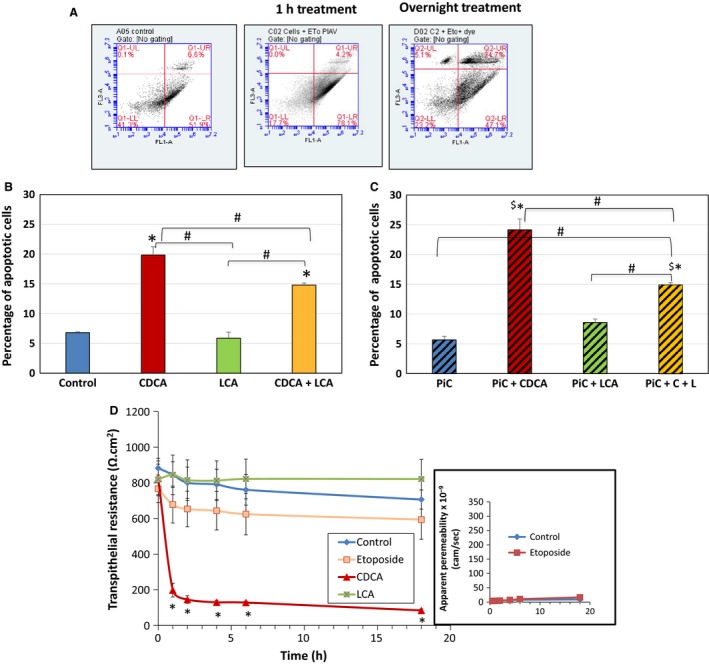
Effects of CDCA (500 *μ*mol/L) and LCA (50 *μ*mol/L) on apoptosis and transepithelial resistance (TER). T84 cells were grown to confluency on six‐well plates and incubated at 37°C with DMSO, 500 *μ*mol/L CDCA, and 50 *μ*mol/L LCA, alone or in combination (overnight (O/N)) ± PiC; etoposide (10 *μ*mol/L,O/N) was used as a positive control. (A) Representative flow cytometry graphs show the effect of DMSO, 1 h and O/N exposure to etoposide on apoptosis. Bar graphs show quantification of flow cytometry results of the effects of O/N treatment with CDCA, LCA, and CDCA + LCA in the absence (B) and in the presence (C) of PiC. Cells were grown in 24 well transwells and time‐dependent effects of DMSO, CDCA, LCA and etoposide on TER (D) was measured. Shown in inset: effect of etoposide on dextran flux reported as apparent permeability. *P < 0.05, compared with DMSO treatment; ^$^ *P* < 0.05, compared to PiC; ^#^ *P* < 0.05, compared with other treatments; *n* ≥ 3.

We next examined if the CDCA‐induced alterations in tight junction function could be attributed to increases in cytotoxicity as measured by LDH release or to apoptosis as seen by annexin V binding in cells grown on nonpermeable supports. While short‐term exposure had no effect, different from control, longer exposures (>2 h ‐O/N) to higher doses (>250 *μ*mol/L) of CDCA caused a small, 17% increase in cytotoxicity (Fig. 5A). This profile of a modest increase (<20%) in cytotoxicity, is also seen with the other 7*α* dihydroxy bile acid, DCA, and with the taurine conjugates of CDCA (TCDC) and DCA (TDC) (Fig. 5B). Similarly, apoptosis was not affected with 1 h exposure to CDCA (5–500 *μ*mol/L), whereas O/N exposure to 500 *μ*mol/L CDCA caused a 13% increase in apoptosis (Table 1, Fig. 6A–B). We recently published that increasing doses of LCA (5–500 *μ*mol/L) for 1 h, or O/N treatment with 50 *μ*mol/L of LCA, were neither cytotoxic nor induced apoptosis in T84 cells (Ao et al. 2016). In this paper, we show that O/N treatment with varying doses of LCA neither significantly increased LDH release (Fig. 5B) nor apoptosis (Fig. 6B). Interestingly, while cytokines alone had no effect, cells treated with PiC+ CDCA, showed a significant increase in apoptosis (Fig. 6C), not different from the action of CDCA alone (Fig. 6B). In contrast, the effects of LCA ± PiC were not different from control, whereas LCA decreased CDCA ± PiC‐induced apoptosis (Fig. 6C). To further confirm that the CDCA‐induced increases in permeability was not due to its modest effects on apoptosis, we determined if etoposide (10 *μ*mol/L) treatment altered TER and dextran flux. As shown in Figure 6D, O/N treatment with etoposide neither altered TER nor dextran flux as compared to control (DMSO) or LCA‐treated cells. In contrast, CDCA increased dextran flux (Fig. 4A) and similar to the report of Keely et al. (2007) on DCA, CDCA causes a time‐dependent decrease in TER (Fig. 6D). To summarize the barrier function studies thus far, CDCA decreased TER and increased transepithelial dextran flux, effects that were enhanced by PiC. In contrast, LCA alone had no effect on either TER or paracellular permeability. While LCA did not alter CDCA's or PiC's effects on TER, it attenuated their effects on permeability as follows: CDCA>40%, PiC≥80%, and CDCA+PiC by 75%. CDCA also reversed ion selectivity of the monolayer, which was unaffected by LCA (Fig. 2). Furthermore, the 20% changes in cell death caused by etoposide are not accompanied by any change in TER or dextran flux. That and the dilution potential data (Fig. 2) suggest that cell death cannot account for the dramatic increases in TER and paracellular permeability in response to CDCA.

**Table 1:** Dose‐dependent effect of CDCA (1 h) on apoptosis in T84 cells

[CDCA] in *μ*mol/L	Percentage of apoptotic cells
5	5.6 ± 1.0
100	5.8 ± 1.1
250	4.1 ± 0.8
500	5.9 ± 1.0
DMSO	4.9 ± 0.8
Etoposide	4.8 ± 1.0
DMSO (O/N)	6.8 ± 0.15
CDCA 500 (O/N)	19.8 ± 1.4^a^
Etoposide (O/N)	26.2 ± 2.5^a^

T84 cells were grown to confluency on six‐well plates and incubated at 37°C as follows: with increasing doses of CDCA, DMSO, or etoposide (10 μmol/L). Samples which were treated overnight are depicted as O/N. All others were treated for 1 h. The O/N etoposide was used as a positive control.

a *P* ≤ 0.05, compared to DMSO; *n* > 4.

### Intercellular adhesion

**Figure 7:**
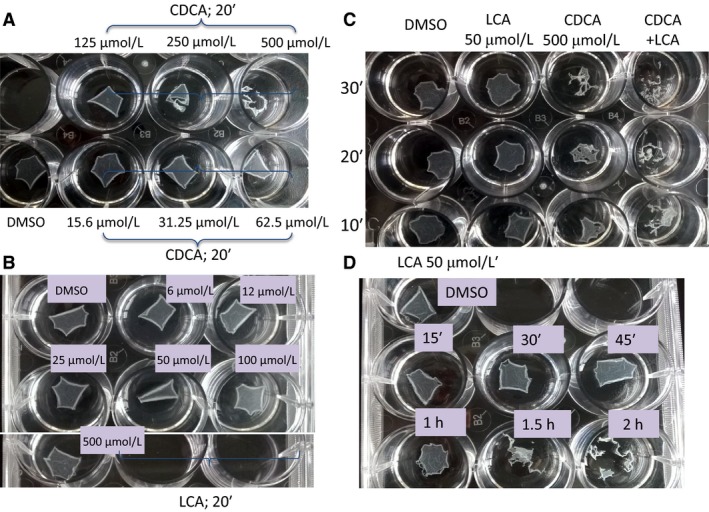
Effects of CDCA (500 *μ*mol/L) and LCA (50 *μ*mol/L) on intercellular adhesion strength as measured by dispase assay. Dispase assay was carried out as described in Materials and Methods. Briefly, confluent T84 cells grown in 12‐well plate were treated with DMSO, CDCA, LCA, or CDCA + LCA for different times before being incubated with dispase‐containing buffer. Representative images show the lifting of the monolayers treated with different concentration of CDCA (A, 20′), LCA (B, 20′), CDCA + LCA (C, 10′–30′), and time course of 50 *μ*mol/L LCA (D); Representative of *n* = 3.

Other intercellular junctional complexes, such as adherens junctions, can also regulate barrier function (Bruewer et al. 2003). Dispase, a protease known to disrupt cell adhesion to the extracellular matrix, has been used to study the effects of agents on intercellular adhesion strength. For example, Jiang et al. (2014) used it to examine the involvement of galectin3 in SKCO15 colonic cells adhesion, where agents that decrease cell–cell adhesion result in fragmentation of the monolayer. To determine if bile acids also affect cell–cell adhesion, we examined the effect of different doses of CDCA and LCA, followed by dispase treatment, on monolayer fragmentation. In DMSO‐treated wells, while dispase treatment detached the monolayer from the substratum, despite agitation, the epithelial sheet remained intact (Fig. 7A). A 20‐min exposure to 16–500 *μ*mol/L CDCA resulted in fragmentation of the monolayer at >250 *μ*mol/L (Fig. 7A). We attempted to count the number of fragments as reported by Jiang et al. (2014). However, in our experience, it was somewhat arbitrary to distinguish between independent fragments and a Swiss cheese cloth‐like appearance of the sheets and therefore the data are presented qualitatively. In contrast to CDCA, a 20‐min exposure to 6–500 *μ*mol/L LCA, did not impact intercellular adhesion (Fig. 7B). While 500 *μ*mol/L CDCA caused monolayer fragmentation even at 10 min (Fig. 7C), 50 *μ*mol/L LCA did not alter the monolayer until exposures of ≥1.5 h (Fig. 7D). When used in combination (Fig. 7C), LCA was unable to attenuate CDCA‐induced monolayer fragmentation. Thus, monolayer fragmentation is enhanced by CDCA > LCA.

### Tight junction proteins

**Figure 8:**
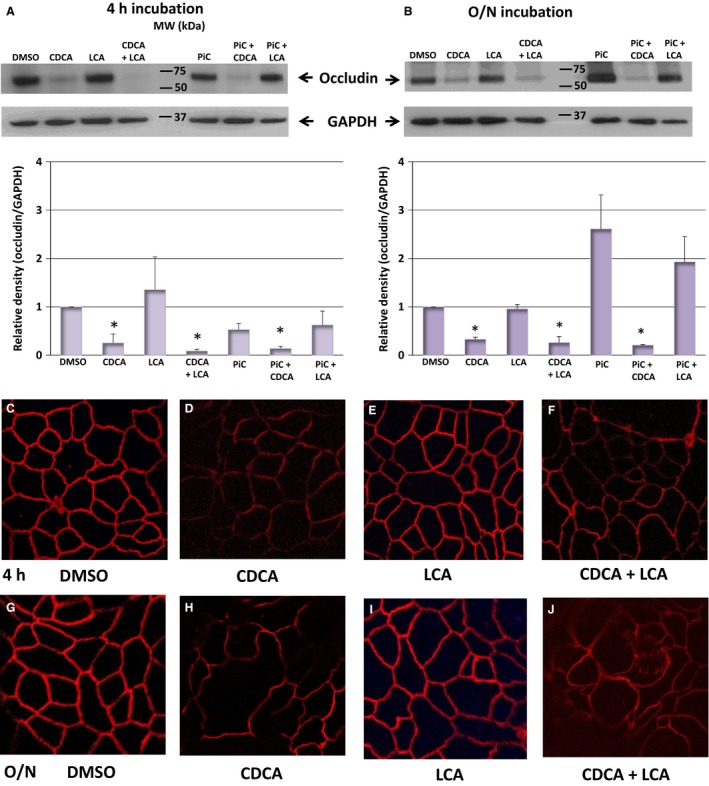
Effects of CDCA (500 *μ*mol/L) and LCA (50 *μ*mol/L) on occludin protein expression and localization. Cells grown on collagen‐coated Transwell inserts were treated apically with DMSO, CDCA, LCA, CDCA + LCA, PiC, PiC + CDCA, or PiC + LCA for 4 h or O/N. Cells were then harvested, and proteins were isolated as described in the Materials and Methods. Representative blots showing occludin expression after 4 h (A) or O/N (B) treatment. Bar graphs show quantitation of the relative density of occludin normalized to GAPDH (A, *n* ≥ 4; B, *n* = 3). *P < 0.05, compared to DMSO. Representative confocal images of CDCA and LCA's effects on occludin localization (red) is shown in C–J. Cells grown on collagen‐coated Transwell inserts were treated apically with DMSO, CDCA, LCA, or CDCA + LCA for 4 h or O/N. Cells were then fixed and stained as described in the Materials and Methods. Magnification: 126x; representative of *n* = 3.

Alterations in a variety of tight junction proteins including occludins, claudins, and the scaffolding protein ZO‐1, (Matter and Balda 2003; Lingaraju et al. 2015), have been correlated with changes in barrier function. With the caveat that there can be many candidate tight junction proteins involved in CDCA action, in this study, we focused on two proteins, claudin‐2 (Lee 2015) and occludin (Raimondi et al. 2008) known to be associated with changes in barrier function in colonic cells. We first determined if exposure to CDCA and LCA altered claudin‐2 and occludin expression by immunoblotting. Surprisingly, with a polyclonal claudin‐2 antibody, we did not find any consistent effect of claudin expression at 2 h (data not shown), 4 h, or O/N exposure to CDCA or LCA ± PiC (Table 2). In contrast, occludin expression, seen by western blots as a single diffuse band of 55–65 kDa (Fig. 8 upper panels) and quantitated in bar graph (Fig. 8 lower panels) was altered by CDCA. Thus, O/N treatment with CDCA caused a decrease (*P* < 0.05) in occludin protein expression that was neither altered by LCA nor by PiC (Fig. 8B). At 4 h, although the mean value was less than that of DMSO, it was not statistically significant. The LCA + CDCA and PiC + CDCA effects were statistically significant both with 4 h and O/N exposure to bile acids. None of the other treatments had a statistically significant effect. In some cases, the mean values appeared large (e.g., LCA at 4 h; PiC ± LCA at O/N) but were not statistically significant, with the number of samples tested.

**Table 2:** Effects of CDCA, LCA, and/or cytokines on claudin‐2 protein expression

		Treatment time	Treatment						
			DMSO	CDCA	LCA	CDCA + LCA	PiC	PiC + CDCA	PiC + LCA
Claudin/GAPDH	Mean ± SEM normalized to DMSO	4 h	1.0 ± 0.0	5.9 ± 4.8^NS^	3.3 ± 2.0^NS^	1.8 ± 1.1^NS^	2.1 ± 1.0^NS^	1.1 ± 0.5^NS^	1.4 ± 0.7^NS^
		O/N	1.0 ± 0.0	0.6 ± 0.1^NS^	2.1 ± 1.2^NS^	3.4 ± 2.8^NS^	6.6 ± 4.2^NS^	3.5 ± 2.8^NS^	4.5 ± 3.0^NS^

Cells grown on collagen‐coated Transwell inserts were treated apically with DMSO, CDCA, LCA, CDCA + LCA, PiC, PiC + CDCA, or PiC + LCA (see Methods for concentrations) for 4 h or O/N. Cells were then harvested, and proteins were isolated as described in the Materials and Methods. Data show relative density of Claudin‐2 expression normalized to GAPDH after 4 h or O/N treatment; NS: not significant; *n* = 3.

We next examined localization of occludin by immunohistochemistry. In control monolayers, the tight junctions revealed a continuous, homogenous occludin ring along the cell circumference in untreated control cells (Fig. 8C), that is disrupted in the presence of CDCA (Fig. 8D) by 4 h (and as early as 2 h; data not shown). Overnight treatment with CDCA results in more prominent discontinuities in occludin distribution (Fig. 8H). Treatment with 50 *μ*mol/L LCA did not reveal a discontinuous pattern in occludin distribution at either 4 h or O/N (Fig. 8E and I, respectively). LCA did not prevent the discontinuities in occludin distribution caused by CDCA (Fig. 8F and J at 4 h and O/N, respectively). In a preliminary immunolocalization study of claudin‐2, we found that the effects of CDCA ± LCA, as in the case of the western blot analyses (Table 2), were equivocal (data not shown) and were not pursued further.

### Cytokine production

**Figure 9:**
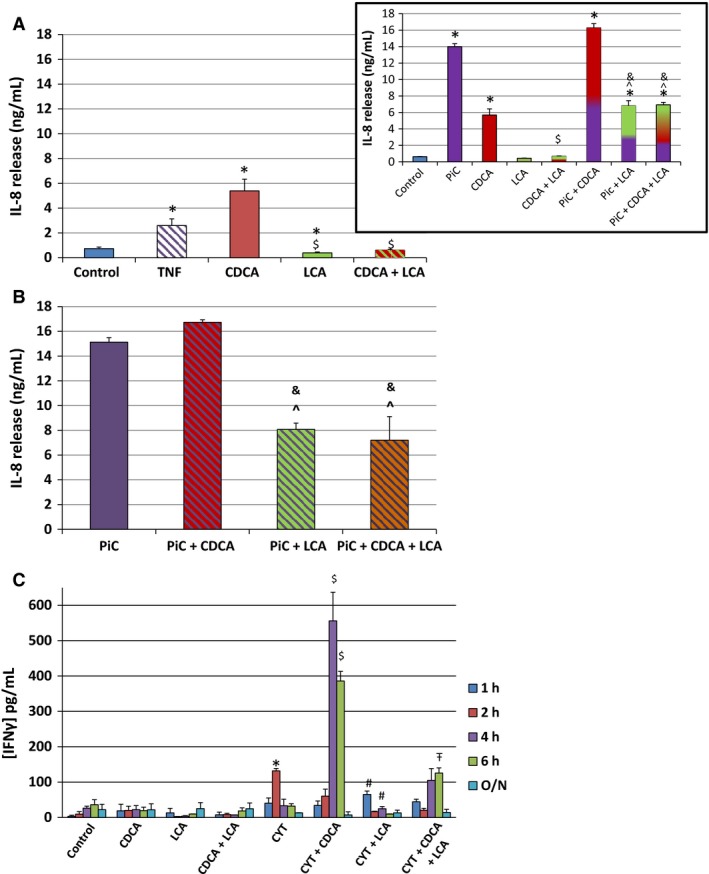
Effects of CDCA (500 *μ*mol/L), LCA (50 *μ*mol/L) and/or cytokines on IL‐8 and IFN *γ* release. Cells were grown to confluency in 24‐well plates or collagen‐coated Transwells, serum‐starved and exposed overnight to DMSO (control), TNF *α*100 ng/mL, CDCA, LCA, or CDCA + LCA O/N (A) and PiC, PiC + CDCA, PiC + LCA, PIC + CDCA + LCA. (B). IL‐8 concentrations in the supernatants (A and B) or apical+basolateral media (inset) were measured using a sandwich ELISA with anti‐human IL‐8 monoclonal antibody. *P < 0.05 compared to control, *n* ≥ 4; ^$^ *P* < 0.05 compared to CDCA; ^*P* < 0.05 compared to PiC; ^&^ *P* < 0.05 compared to PiC+CDCA. (C) IFN *γ* concentrations were measured using anti‐human IFN *γ* monoclonal antibody in supernatants collected from cells treated with DMSO (control), CDCA, LCA, or CDCA + LCA in the presence or absence of CYT ([TNF *α* [10 ng/mL] + IL‐1*ß* [10 ng/mL]]). *P < 0.05, compared to CYT at 1 h, 4 h, 6 h or O/N or control; ^$^ *P* < 0.05, compared to CYT+CDCA at 1 h, 2 h, and O/N; ^#^ *P* < 0.05, compared to CYT+LCA at 2, 4, 6 h and O/N; *n* ≥ 4 each run at least in duplicate.

The proinflammatory cytokines TNF*α* and IFN*γ*, commonly found in IBD patients, are known to downregulate occludin expression in Caco‐2 and T84 cells and alter barrier function (Zolotarevsky et al. 2002). Further, our studies (Fig 1B and 4; (Ao et al. 2013)) show that these cytokines, with IL1*ß* (PiC), decrease TER and increase dextran flux. Therefore, we examined if cytokines mediate the effect of bile acids on barrier function in T84 cells. We have previously shown in T84 cells that TNF*α* induced the release of IL‐8, another proinflammatory cytokine known to alter epithelial integrity (Boonkaewwan et al. 2008). We probed if bile acids caused the release of IL‐8, in confluent T84 cells grown on six‐well plastic dishes as well as those grown on permeable Transwell supports. The cells were serum‐starved O/N and IL‐8 ELISA was performed in cells treated (O/N) with CDCA ± LCA ± PiC or TNF*α* (100 ng/mL) (see methods). When grown on plastic, while TNF*α* and CDCA, respectively, caused four‐ and fivefold increases in IL‐8 production, LCA caused a 50% reduction in basal and an 83% decrease in CDCA‐induced IL‐8 production (Fig. 9A). The inflammatory mediator cocktail, PiCs stimulated IL‐8 release ~21‐fold, (Fig. 9B) four times greater than that seen with CDCA, and this was not further significantly enhanced by CDCA (~23‐fold); however, LCA reduced PiC ± CDCA response by 50% (Fig. 9B). Equally important, CDCA, LCA, and PiC, elicited remarkably similar IL‐8 responses when cells were grown on permeable supports (Fig. 9 inset), suggesting that in terms of IL‐8 production, the bile acids and PiC have ready access to the surfaces of the monolayer regardless of the substratum. Overall, the results with IL‐8 parallel the inhibitory effects of LCA on CDCA ± PiC‐induced increases in 10 kDa dextran flux (Figs. 3 and 4).

We next determined if TNF*α* is an intermediary in CDCA‐induced IL‐8 release. Our studies showed that neither CDCA nor LCA increased TNF*α* release in T84 cells (Table 3). The validity of the assay was corroborated by the fact that 6 h exposure to IFN*γ* caused *a* > 10‐fold increase in TNF*α* release from RAW 267.4 macrophages (TNF*α* pg/mL: Baseline: 22.8 ± 5.2; +IFN*γ* (10 ng/ml): 280 ± 10; *n* = 3).

**Table 3:** Dose‐dependent effects of CDCA and LCA on TNFα release in T84 cells

[TNF*α*] in pg/mL		
Concentration (*μ*mol/L)	Treatment	
	CDCA	LCA
DMSO	9.4 ± 1.7	9.2 ± 1.4
5	8.7 ± 1.4^NS^	12.7 ± 2.0^NS^
50	8.0 ± 0.7^NS^	11.7 ± 1.0^NS^
100	10.2 ± 1.4^NS^	10.5 ± 1.7^NS^
250	10.9 ± 1.1^NS^	11.9 ± 1.7^NS^
500	9.1 ± 1.0^NS^	9.8 ± 1.8^NS^

Dose‐dependent effects of CDCA and LCA on TNF*α* release in T84 cells. Cells were grown to confluency in 96‐well plates and TNF*α* was measured using anti‐human TNF*α* monoclonal antibody (BD Biosciences), in supernatants of serum‐starved (O/N) cells treated with increasing doses of CDCA and LCA; *n* = 4, each run in triplicate.

NS, No significant increase in [TNF] over control.

Finally, IFN*γ* production was significantly stimulated at 4 and 6 h, by CDCA in combination with a cocktail of TNF*α* and IL1ß, and surprisingly CDCA alone had no effect at any time point (Fig. 9C). Interestingly, at a single time point, 2 h, the TNF*α* and IL1ß cocktail stimulated IFN*γ* release. As seen with IL‐8 release, LCA was able to attenuate the effects of cytokines±CDCA on IFN*γ* release (Fig. 9C). Thus, CDCA and LCA also have opposing effects on the production of selective cytokines.

### Role of oxidative stress and ROS in bile acid action

**Figure 10:**
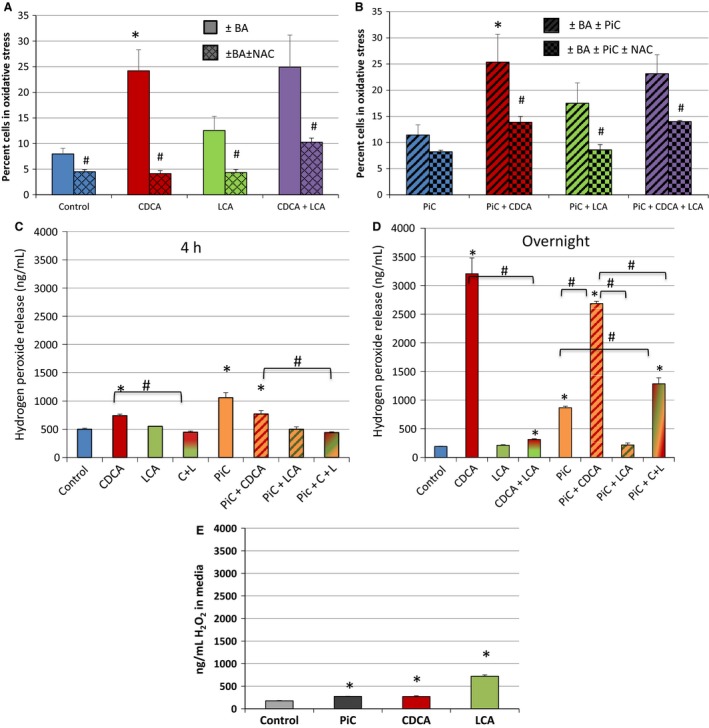
Effects of CDCA, LCA, and/or PiC on reactive oxygen species production. T84 cells grown in 24‐well plates were serum‐starved and treated with O/N with DMSO, CDCA, LCA, or CDCA + LCA ± N‐acetyl cysteine (NAC; 1 mmol/L, scavenger of ROS) in the absence (A) or presence of PiC (B). Mitochondrial and nuclear ROS production in cells stained with CellRox Green and Systox Red (stains dead cells) was measured using flow cytometry and reported as percent of cells that are CellRox positive. T84 cells grown in 24‐well plates were serum‐starved and treated with DMSO (control), CDCA, LCA, or CDCA + LCA for 4 h (C) or O/N (D), in the presence or absence of O/N PiC. Hydrogen peroxide (H_2_O_2_) in the cell culture supernatant was measured using a colorimetric detection kit as described in the Materials and Methods. RAW 264.7 cells (~20,000) were treated with PiC, CDCA (500 *μ*mol/L), or LCA (50 *μ*mol/L) for 4 h and culture supernatants were assayed to measure H_2_O_2_ release (E). *P < 0.05 compared to control; ^#^ *P* < 0.05, compared as indicated; *n* ≥ 4.

ROS has been shown to be involved in bile‐acid alteration of Caco‐2 cell permeability (Araki et al. 2005) and in bile acid stimulation of rat colonic epithelial cell proliferation (Craven et al. 1987). Thus, we examined the effect of CDCA ± LCA, and ±PiCs on ROS production using two approaches. First, nuclear and mitochondrial ROS production was assessed by staining with CellROX Green and flow cytometry. As shown in Figure 10A and B (nonhatched bars), CDCA ± PiC increase ROS production and LCA does not attenuate either of these increases. Neither LCA nor PiC alone significantly altered ROS production as compared to DMSO control. To determine the specificity of CDCA action, the effect of the ROS scavenger, NAC (1 mmol/L, determined to be optimal concentration, data not shown) was examined. As shown in the cross‐hatched bars, NAC decreased ROS in all the samples. This method is qualitative in that it detects ROS‐positive cells and neither quantitates the amount of ROS produced nor accounts for ROS released from the cells.

Therefore, the metabolic by‐product of ROS, hydrogen peroxide (H_2_O_2_), was measured in cell culture supernatants of T84 cells after 4 h and O/N exposure to various bile acid treatments. As shown in Figures 10C and D, there were dramatic time‐dependent differences in ROS production. At 4 h, both CDCA, and PiC, alone or in combination caused modest 1.5–2‐fold increases in H_2_O_2_ release, which were attenuated by LCA, with LCA alone having no effect (Fig. 10A). With O/N exposure, both control and LCA‐treated samples were similar, but showed a 50% decrease as compared to the 4 h time point. While O/N PiC alone showed a modest further increase (~4.5‐fold) in H_2_O_2_ production, CDCA (~17‐fold) and CDCA+PiC (~14 fold) showed dramatic increases in ROS production. While LCA completely attenuated the CDCA effect, it only partially inhibited (~50%) the oxidative stress caused by CDCA±PiC (Fig. 10B). Likewise, CDCA and PiC increased ROS production (~2‐fold) in RAW 264.7 macrophages. However, in marked contrast to T84 cells, LCA caused a dramatic (~4‐fold) increase in ROS production in the macrophages, suggesting cell‐specific effects of bile acids (Fig. 10E).

**Figure 11:**
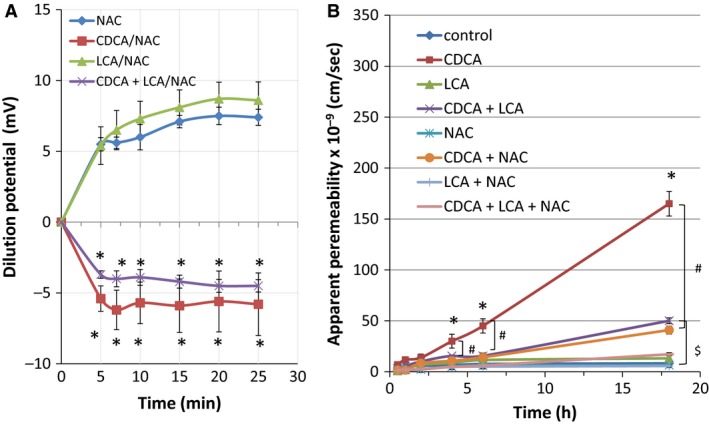
Effects of ROS scavenger, NAC, on CDCA and LCA induced changes in ion selectivity and 10 kDa dextran flux. Cells grown on collagen‐coated Transwell inserts were used to measure dilution potentials (D*ψ*) as described in Fig. 2. (A). Briefly, the initial *ψ* was recorded with Buffer A (containing 120 mmol/L NaCl) in the apical chamber, using chopstick electrodes and EVOM voltohmmeter. Then the Buffer A was replaced with Buffer B (containing 60 mmol/L NaCl and 120 mmol/L mannitol) and *ψ* was recorded until it reached a steady level (25 mins). Buffer B contained DMSO (control), CDCA (500 *μ*mol/L), LCA (50 *μ*mol/L), or CDCA + LCA, in the presence or absence of NAC. Dextran flux was measured as described in Figure 4. Cells were serum‐starved overnight in the absence or presence of PiC ± NAC and an apical exposure (AM) to DMSO, CDCA (500 *μ*mol/L), LCA (50 *μ*mol/L), or CDCA + LCA. Dextran flux was expressed as apparent permeability (cm/sec), as described in the Materials and Methods. *P < 0.05 vs. control; ^#^ *P* < 0.05 vs. CDCA + NAC; ^$^ *P* < 0.05 vs. CDCA + LAC + NAC,* n* ≥ 4.

Finally, we determined if CDCA‐induced increases in ROS was indeed responsible for CDCA's action on barrier function. We examined if scavenging ROS with NAC would affect CDCA's effect on pore and gate functions. In terms of pore function, NAC neither altered CDCA±LCA‐induced decreases in TER (% decrease compared to DMSO; 1H CDCA: 83 ± 7; CDCA+NAC: 84 ± 5; CDCA+LCA: 80 ± 3; CDCA+LCA+NAC: 81 ± 7, *n* = 3) nor ion selectivity as measured by DΨ (Fig. 11A). Further, NAC had no significant effect on BA‐induced apoptosis (18 H: DMSO 8 ± 0.3%, CDCA: 18 ± 1%; LCA: 8 ± 0.2%; CDCA+LCA: 12 ± 0.3%; NAC: 9 ± 0.4%; CDCA+NAC: 15 ± 1%; LCA+NAC: 7 ± 0.1%; CDCA+LCA+NAC: 14 ± 0.2%, *P* > 0.05, *n* = 6). In contrast, NAC altered the leak function over time causing ~65% reduction in CDCA‐induced dextran flux (Fig. 11B). As noted in Figure 4, (18 h, *μ*g of F10D: Control: 8 ± 1; CDCA: 165 ± 12; NAC: 6.5 ± 4; CDCA + NAC: 41 ± 2; *P* < 0.05, *n* ≥ 4, LCA diminished (by 60%) CDCA's action on dextran flux and this was further attenuated by NAC (18 h (*μ*g): LCA: 13 ± 2; NAC + LCA: 6 ± 0.2; CDCA + LCA: 50 ± 3; NAC + CDCA + LCA: 17 ± 1; *P* < 0.05, *n* ≥ 3) (Fig. 11B). We demonstrate that ROS plays a role in CDCA‐induced TJ dysfunction by altering leak, but not pore function in T84 cells.

## Discussion

Bile acids are increasingly recognized as signaling and regulatory molecules and can act via nuclear or membrane‐bound receptors to affect a variety of physiological functions in the gastrointestinal tract as well as systemically, and perturbation of these functions can lead to disease. For example, excess bile acids can cause secretory diarrhea (Binder 2006; Alrefai and Gill 2007; Islam and DiBaise 2012) and might play a role in intestinal inflammation seen in chronic diseases such as Crohn's and ulcerative colitis (Pavlidis et al. 2015). Various investigators, including our group (Mekjian et al. 1971; Chadwick et al. 1979; Martinez‐Augustin and Sanchez Medina 2008; Bajor et al. 2010; Ao et al. 2013; Domingue et al. 2016), have reported that the 7*α*‐dihydroxy bile acids CDCA and DCA stimulate colonic fluid and electrolyte secretion, whereas the monohydroxy LCA can attenuate secretagogue‐stimulated secretion (Ward et al. 2013; Ao et al. 2016). While Cl^−^ secretion is largely a transcellular process, changes in epithelial permeability via the paracellular pathway can also affect transport functions. Individual bile acids, as well as proinflammatory cytokines (PiC) have been reported to increase colonic epithelial permeability (Chadwick et al. 1979; Bruewer et al. 2003; Araki et al. 2005; Munch et al. 2007; Raimondi et al. 2008; Vitek 2015), but there have been fewer studies comparing the long‐term effects of different bile acids added alone or in combination.

Thus, we studied the effects of long‐term treatment of CDCA and LCA, in the presence or absence of PiC, on tight junction function as measured by alterations in TER, paracellular macromolecular permeability, pore charge selectivity, adhesion strength, tight junction proteins, cytokine, and ROS production. To the best of our knowledge, this study is the first to demonstrate that CDCA and LCA have dissimilar effects on barrier gate functions, with CDCA dramatically altering it and LCA having no direct effect. Equally important, LCA attenuates select, but not all of CDCA's actions. CDCA alters pore and leak functions, and both effects were enhanced by PiC. CDCA also altered the charge selectivity and decreased intercellular adhesion, while LCA had no effect ± CDCA on these or on the pore functions. In contrast, LCA partially restored the leak function that was disrupted by PiC ± CDCA. CDCA plays a role in the inflammatory response, enhancing the release of IL‐8 and ROS, and augmenting the effects of TNF*α* and IL1‐*ß* on IFN*γ* production, while LCA suppresses these actions. Like LCA, scavenging ROS had no effect on the pore function, but attenuated CDCA‐induced increases in leak pathway.

The physiological relevance of these complex interactions have to be examined in the context of our current knowledge of the paracellular pathway. Over the last 30 years, it has been firmly established that the space between cells in epithelia is a dynamic compartment, with flexible geometry governed by a series of junctional complexes starting near the lumen with the zona occludens (ZO), followed by the zona adherence, desmosomes, and gap junctions; finally, hemidesmosomes help the epithelial cell adhere to the basement membrane. Complex protein–protein, lipid–lipid and protein–lipid interactions undergird these junctions with the membrane proteins also being tethered to, and influenced by, the lattice of intracellular cytoskeletal, scaffolding, and regulatory proteins. It is well established that disruption in barrier function plays a key role in the development of intestinal inflammatory diseases (Groschwitz and Hogan 2009; Peterson and Artis 2014; Vitek 2015); and in the etiology of a number of enteric bacterial infections (Viswanathan et al. 2009). Proinflammatory cytokines, inflammatory mediators, and bacterial toxins can cause both structural reorganization and functional disruption of the barrier (Vitek 2015).

Recently, there has been a resurgence of interest in the role lipid interactions between adjacent bilayers may play in defining paracellular movement (Farquhar and Palade 1963; Lingaraju et al. 2015). Bile acids are surfactants and disrupt the barrier at millimolar concentrations. For example, DCA and CDCA (5–20 mmol/L), reversibly increase intestinal permeability in the rat (Sun et al. 2004a,b), the rabbit (Chadwick et al. 1979), and the pig (Henrikson et al. 1989). In contrast, 5 mmol/L CA and UDCA had no effect in the rabbit colon (Chadwick et al. 1979). The concentrations of CDCA (500 *μ*mol/L) and LCA (50 *μ*mol/L) used in this study, selected for their known effects on ion transport, are well below their CMC (vide supra*,* Introduction). Therefore, it is unlikely that the observed effects are due to their detergent properties. It is also unlikely that the effects of CDCA on the pore and leak pathways are due to the small increase in apoptosis (≈13%) or cytoxicity (≈20%) seen at 18 h, for the following reasons: First, the positive control for apoptosis, etoposide, neither altered TER nor dextran flux (Fig. 6D); second, the change in ion selectivity caused by CDCA is more likely due to a change in the tight junction structure than a loss of cells due to apoptosis; third, while CDCA ± PiC increased permeability of 10 kDa dextran, it did not increase permeability to 70 kDa dextran (data not shown) and fourth, the D*Ψ* changes are seen at a time where there is no change in cytotoxicity or in apoptosis (Fig. 5, Table 1). While LCA, at different concentrations, has been reported to be toxic to hepatocytes (Beilke et al. 2008), it neither altered the percent cytotoxicity nor apoptosis in T84 cells (Fig. 5B). In contrast, LCA decreased (40%) the apoptosis seen in the presence of CDCA ± PiC. To the best of our knowledge, a role for low doses of LCA as an antiapoptotic agent has not yet been explored.

Our data clearly indicate that the two bile acids we studied have very different effects on the gate function of paracellular movement, with CDCA causing drastic alterations in both the pore and leak properties, whereas LCA alone did not affect either function. CDCA caused a dramatic, dose‐dependent reduction in TER (Figs. 1A and 6D), generally a measure of pore function, and LCA had no effect. As reported by us (Ao et al. 2013) and others (Bruewer et al. 2003; Groschwitz and Hogan 2009), PiC also caused a decrease in TER, although this was not as dramatic as that caused by CDCA (Fig. 1). While not a given, alteration in TER could also result in an alteration of the ion selectivity of the pathway (Van Itallie et al. 2003; Van Itallie and Anderson 2006). This is indeed the case in our study since CDCA, but not LCA, reversed the *D*Ψ across the T84 monolayers, shifting it from a cation‐selective to an anion‐selective profile (Fig. 2). Equally important, LCA did not alter the effects of either CDCA or PiC on TER or of CDCA on D*Ψ*, suggesting that it has no modulatory effects on pore function. These results are intriguing considering that anion and cation selectivity have been ascribed to specific claudins (Van Itallie et al. 2003; Van Itallie and Anderson 2006; Lu et al. 2013; Lingaraju et al. 2015) and are discussed in further detail below.

CDCA also altered the leak function of the T84 monolayers, causing a time‐dependent increase in the permeability of 10 kDa dextran, greater than that seen with DMSO alone. This was observed only when CDCA was applied to the AM of the monolayer, as addition to the BLM was not different from that of DMSO. Not surprisingly, PiC alone increased the leak function, and this was augmented by CDCA in the AM. The kinetics of the PiC + CDCA effect appears biphasic (Fig. 4B), but in examining the absolute values it appears that the effects of CDCA and PiC are additive at both 8 h and 18 h (Fig. 4A vs. B). LCA alone had no effect on the leak function, but interestingly, it caused a 50% reduction in the CDCA‐induced increases in permeability, and a more robust, ~80% reduction in the PiC alone or CDCA+PiC responses. It remains to be determined if this is due to different signaling mechanisms being involved in the interfacing of the actions of CDCA, PiCs, and LCA and/or simply that higher doses of LCA would cause a total inhibition of the CDCA action.

Although there are more than 50 different proteins associated with the ZO region, most attention has been paid to the claudins, occludins, and the ZO‐1 proteins (Matter and Balda 2003; Anderson and Van Itallie 2009; Lingaraju et al. 2015). Claudins have been chiefly associated with the pore function and are important in determining size (for ions and solutes up to 3.5 Å) and charge selectivity and appear to have little, if any role to play in the leak pathway (Van Itallie et al. 2003; Van Itallie and Anderson 2006; Lingaraju et al. 2015). For example, claudin‐2 is associated with conferring cation selectivity of the pore pathway of epithelial layers (Luettig et al. 2015). In contrast, occludin distribution is linked to changes in the leak pathway (Rao 2009). Thus, agents that increase epithelial permeability cause a decrease in the junctional localization of occludin, often by endocytosis (Marchiando et al. 2010; Lingaraju et al. 2015).

While considerable attention has been paid to the structure and function of the ZO region, recent evidence indicates that other regions, like desmosomes, may play a role greater than just cell–cell adhesion. Therefore, although the movement of substances via the paracellular pathway is in response to passive driving forces, it is nevertheless strongly influenced by the geometry and selectivity of the macromolecules lining the pathway.

To determine the molecular basis of the changes in paracellular permeability, it is not possible to screen the vast array of junctional proteins and we selected claudin‐2 and occludin, as the most likely candidates, based on an excellent body of work, some of which is summarized below. In general, a decrease in TER is often associated with an increase in claudin‐2 expression and an increase in permeability is associated with a decrease in the expression and/or tight junction localization of occludin. Of the 26 human isoforms of claudin, claudin‐2 is upregulated in the small and large intestine during inflammatory conditions as seen in Crohn's disease and colitis and been shown to play a role in barrier dysfunction leading to diarrhea (Luettig et al. 2015). Mucosal biopsy samples of patients with ulcerative colitis showed ~955% increase in claudin‐2 expression (Lu et al. 2013). Claudin‐2 forms a channel that is cation selective allowing small ions to pass through, thus forming a size‐ and charge‐selective pore pathway (Van Itallie and Anderson 2006). Inflammatory cytokines either upregulate claudin‐2 expression and/or alter its phosphorylation or sumoylation states. IL‐6, a proinflammatory cytokine increased tight junction permeability and increased claudin‐2 expression in Caco‐2 cells. In T84 cells, IL‐17 increased claudin‐2, not claudin‐1, expression via a MEK pathway (Lee 2015), while IL‐13 increased claudin‐2, but not claudin 3 and 4 expression, and increased paracellular permeability (Prasad et al. 2005). T84 cells (Fig. 2), like the MDCK cell line (Van Itallie et al. 2003) are cation selective and the CDCA‐induced loss of cation selectivity (Fig. 2), would have been consistent with an alteration of claudin‐2 distribution. However, despite the compelling background for a central role for claudin‐2 in TER and cation selectivity, the CDCA‐induced changes in pore function in T84 cells are neither due to alterations in claudin‐2 protein expression at 4 h or O/N (Table 2), nor to its redistribution (preliminary observations, not shown). While these results were surprising, it is conceivable that the alterations in pore function could be due to posttranslational phosphorylation or sumoylation or involvement of other members of the claudin family. For example, intestine‐specific deletion of claudin‐7 results in increased permeability to small organic solutes (Tanaka et al. 2015), and in a maternal immune activation model, increased permeability is associated with a decrease in claudin‐8 and an increase in claudin‐15 expression (Gunzel and Yu 2013).

Although some studies in occludin knock‐out mice suggest that the role for occludin in tight junctions is controversial (Saitou et al. 2000; Oshima and Miwa 2016), there is considerable evidence linking alterations in occludin expression with the leak function. For example, overexpression of occludin increased TER in MDCK cells (Balda et al. 1996) and decreases TNF*α*‐induced barrier dysfunction (Marchiando et al. 2010). Furthermore, occludin rearrangement and dephosphorylation was associated with CDCA (50 *μ*mol/L)‐induced transient increases in permeability in Caco‐2 cells (Raimondi et al. 2008). In contrast to the lack of effect on claudin‐2, CDCA caused a reduction in the expression of occludin that was not altered by LCA (Fig. 8A and B). PiC or LCA alone did not significantly alter occludin expression and they were unable to prevent the effect of CDCA on decreasing occludin expression. Immunolocalization studies (Fig. 8C–J) corroborate the western blot data and revealed a disruption of occludin distribution by CDCA±LCA. The CDCA action on occludin redistribution is consistent with the dextran flux data. However, the ability of LCA to only attenuate the CDCA‐induced increase in dextran flux, but not the decrease in occludin expression or localization, leads to the speculation that LCA may be acting via other steps of the leak pathway. These could include ZO‐1, JAM, other scaffolding proteins, and/or the signaling cascade of MLCK and the contractile proteins of the terminal web (Cunningham and Turner 2012; Lingaraju et al. 2015). Exploring the roles of other claudins and other tight junction proteins and bile acid‐induced regulation of these pathways will be the focus of future studies.

Our results also indicate that complexes other than the tight junctions could contribute to the actions of CDCA on paracellular permeability. At early time points (<1 h), CDCA, in a dose‐dependent manner, decreased the adhesion, fragmenting the monolayer, whereas LCA altered intercellular adhesion only at later time points (Fig. 7). As in the case of pore function, LCA did not alter the effects of CDCA on decreasing intercellular adhesion. The molecular basis of these effects remains to be determined. Two intriguing possibilities are desmogleins and their binding partners galectins (Jiang et al. 2014), and claudin‐7, which is known to have a non‐tight junction role in promoting epithelial cell attachment (Lu et al. 2015).

Our observations with PiC exemplifies the well‐documented regulation of barrier function by inflammatory mediators (e.g., (Zolotarevsky et al. 2002; Bruewer et al. 2003; Muhlbauer et al. 2004; Capaldo and Nusrat 2009; Pavlidis et al. 2015)). Here, we report that both cytokines and ROS appear to be involved in CDCA action (Figs. 9, 10). Equally relevant, our data suggest that LCA has both an anti‐inflammatory and antioxidant role in T84 cells. The interactions of immune modulators are complex and therefore it is no surprise that while there are commonalities in PiC and CDCA effects, there are distinct differences. What is apparent is that LCA attenuates (≥50%) or completely mitigates CDCA ± PiC action on cytokine and ROS generation. The data in Figure 9A, showing a CDCA‐stimulated increase in IL‐8 and its reversal by LCA, make it tempting to postulate a regulatory role for IL‐8 in the leak pathway. The data in Figure 9B on PiC±CDCA having a dramatic effect on IL‐8, and the attenuation by LCA, corroborate such a postulate. It must be cautioned that IL‐8 alone may not be responsible for the increase in dextran flux, since although PiC>>CDCA increases IL‐8 production, the effects of PiC on dextran flux are lower than that of CDCA (Fig. 4B vs. A). Our data in Figure 9 (inset) show that IL‐8 production is not altered by the matrix (plastic vs. transwells) on which the cells are grown, while other groups have examined the influence of only one type of substratum on function (Schuerer‐Maly et al. 1994; Li et al. 1998) However, it is conceivable, that depending on the signaling step being examined (e.g., ROS vs. cytokines) the substratum could influence the structure (Madara et al. 1987) and/or the response and this needs to be considered as this signaling cascade is unraveled in future studies.

The signaling steps leading from CDCA to IL‐8 remain to be determined, but thus far, in T84 cells, the direct effect of CDCA does not involve an increase in TNF*α* (Table 3) or IFN*γ* (Fig. 9C), both of which are known to stimulate IL‐8 in this (Boonkaewwan et al. 2008) and other cell types (Eckmann et al. 1993; Kolios et al. 1996). What is clear is that LCA abolishes the effect of CDCA on IL‐8 production. Our TNF*α* data (Table 3) suggest that either T84 cells do not produce TNF*α* or that the latter is not involved in CDCA‐induced IL‐8 release or that another intermediary signal is required. TNF*α* generation is complex and, for example, CDCA > UDCA directly inhibit endotoxin‐induced release of TNF*α* by macrophages in the liver (Greve et al. 1989; Calmus et al. 1992). While CDCA alone has no effect, TNF*α* and IL‐1ß increase IFN*γ* transiently at 2 h, and in combination with CDCA they synergistically increase IFN*γ* production at 4 and 6 h. These observations suggest that CDCA may invoke IFN*γ* only under inflammatory conditions. Equally relevant, the effects of (TNF*α *+ IL‐1ß) ± CDCA are attenuated by LCA. This temporal pattern of IFN*γ* should be viewed in context of whether it is an early trigger of ROS production as well as having a dual role both as a proinflammatory cytokine and with anti‐inflammatory effects (Muhl and Pfeilschifter 2003; Wisner et al. 2008). Overall, it will be important to consider the temporal patterns of modulator expression when determining the sequence of CDCA and LCA signaling.

The generation of ROS by the conversion of hydrogen superoxide to H_2_O_2_, has been linked to barrier dysfunction and many inflammatory diseases (Banan et al. 2000). In Caco‐2 monolayers, ROS generation has been implicated in CA‐induced drop in TER (Araki et al. 2005). In rat hepatocytes (Sola et al. 2002) and BCS‐TC2 cells (Barrasa et al. 2013), bile acids induced apoptosis by causing mitochondrial disruption and alterations in oxidative phosphorylation resulting in ROS generation (Barrasa et al. 2013). Our studies show that CDCA‐induced oxidative stress as measured by H_2_O_2_ production in T84 cells is even more prominent than that induced by PiC‐ or PiC+CDCA (Fig. 10C). Perhaps this is because CDCA and PiC use different signaling mechanisms to activate different ROS generators such as xanthine oxidase or NADH dehydrogenase, as reported by Araki et al. (2005). In marked contrast, LCA had an antioxidant role by completely attenuating the CDCA‐induced increase in H_2_O_2_ release from T84 cells. Equally important is the demonstration that the ROS scavenger, NAC, like LCA, attenuates CDCA‐induced dextran fluxes but fails to alter CDCA‐induced changes in TER or ion selectivity. It is therefore tempting to postulate that LCA could be maintaining barrier integrity by preventing ROS release. However, not all actions of LCA can be attributed to ROS, since only LCA (Fig. 6), but not NAC, partially attenuates the effects of CDCA on apoptosis.

Interestingly, LCA had the opposite effect in RAW 264.7 macrophage cells, where it stimulated ROS more robustly than PiC and CDCA. This cell specificity could be due to differences in signaling cascades. Thus, macrophages express high levels of the bile acid receptor TGR5 (Lou et al. 2014) and its activation mediates bile acid‐induced IL‐1ß and TNF‐*α* expression via JNK‐dependent pathway. In contrast, we have shown that LCA does not induce the release of TNF*α* (Table 3), does not increase cAMP in T84 cells, and its action in attenuating cAMP‐mediated Cl^−^ secretion is not via TGR5 (Ao et al. 2016).

This segues into the question of how do CDCA and LCA act on T84 cells to cause changes in barrier function? Briefly, bile acids can act via the nuclear receptors, FXR or VDR, or membrane‐bound receptors including TGR5, muscarinic M3 receptors, and sphingosine receptors, to name a few (Li and Chiang 2013). We have explored the signal transduction cascades undergirding the short‐term effects of CDCA on Cl^−^ transport in T84 cells and demonstrated that it involves cAMP, Ca^2+^, and EGFR signaling, with considerable cross‐talk. These data also suggested that neither LCA nor CDCA appear to use the well‐recognized nuclear (FXR, VDR) or membrane (TGR5, muscarinic M3) bile acid receptors to affect Cl^−^transport. Interestingly, while both bile acids activated the MAPK‐related ERK and p38 pathways, the specific MEK and p38 kinase inhibitors failed to attenuate CDCA or LCA's actions on Cl^−^ transport, underscoring the importance of defining the biological end point when studying signaling cascades. Although p38 is known to be involved in a number of stress responses (Obata et al. 2000), we found that the CDCA effect on monolayer disintegration in the adhesion assay was not attenuated by the p38 inhibitor SB208530 (10 *μ*mol/L, *n* = 4; data not shown). We are cognizant that until we undertake a detailed analysis of receptor and second messenger cascades underlying the long‐term changes in paracellular permeability, any conclusions will be speculative at best.

Studies in reductionist models, such as we have employed in T84 cells, provide useful clues in our understanding of pathophysiological mechanisms. Thus, in considering the actions of dihydroxy bile acids such as CDCA in inflammation and diarrhea in vivo, the ameliorating effects of lower concentrations of LCA should be taken into account, especially since the luminal concentrations of LCA in the colon are far lower than that of CDCA (Hofmann and Mysels 1992).

**Figure 12:**
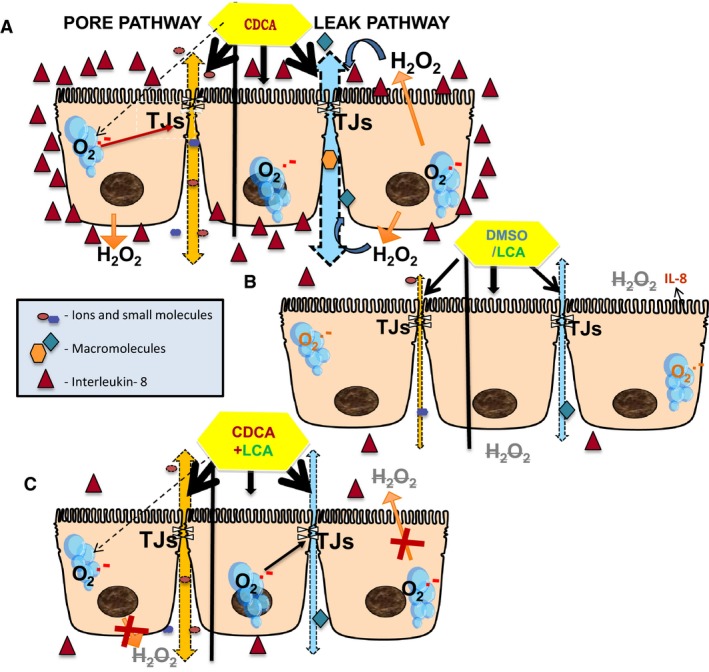
Summary. Panel A: CDCA dramatically alters barrier function, by increasing both pore and leak functions. CDCA increases production of the proinflammatory cytokine, IL‐8, and induces oxidative stress as shown by increases in mROS and H_2_O_2_ release. ROS plays a role in the leak but not pore pathway. Panel B: LCA neither alters barrier function, nor increases IL‐8 production nor induces oxidative stress. Panel C: LCA does not alter CDCA's actions on pore function. LCA partially attenuates CDCA's actions on the leak function, on IL‐8 release and H_2_O_2_ release. Proinflammatory cytokines exacerbate CDCA action and LCA attenuates these effects on leak function, IL‐8, and H_2_O_2_ release (not shown in model).

When considering our results in a temporal sequence, an early response to CDCA is a shift in the ion selectivity of the T84 monolayer from cation selective to anion selective. This is followed by a drop in TER that is sustained up to 18 h. These alterations in pore function can be enhanced by PiC, a scenario that could exist under inflammatory conditions. The drop in TER over the first hour is also accompanied by a loss of intercellular adhesion. Neither the baseline parameters nor the actions of CDCA on these pore functions are altered by LCA. The increase in paracellular permeability could allow the luminal bile acids to access the BLM, thus providing an explanation for the hitherto unanswered question of how although present in the lumen, bile acids are more effective in stimulating ion transport changes from the basolateral surface. With time (>4 h) CDCA ± PiC increase macromolecular permeability, enhance cytokine and ROS production, and decrease occludin expression. With the exception of occludin expression, these leak functions are attenuated by low concentrations of LCA. It is tempting to postulate that while CDCA causes an increase in the pore function, its leak functions are curtailed by the presence of LCA, which may be acting as a scavenger and suppressing the release of both cytokines and ROS (Fig. 12). In addressing pore and leak functions our data also suggest that intercellular junctions, other than tight junctions, should also be considered when examining paracellular permeability. Bile acids can alter intestinal function by regulating motility (Pattni and Walters 2009), gut‐associated immune function and/or as we have discussed, trans‐ or paracellular transport across epithelia. Therefore, in vivo, our postulates need to be tested against a backdrop of a myriad factors, ranging from the relative concentrations of the bile acids and the biophysical (surfactant) properties thereof, to the contributions of other cell types including GALT, the mesenchyme, musculature and last, but not least, the luminal microbiome (Viswanathan et al. 2009; Hofmann and Hagey 2014; Van Itallie and Anderson 2014). In addition to influencing the expression of various proteins involved in bile acid metabolism, the gut microbiome contributes to the relative distribution of individual bile acid species by regulating 7‐dehydroxylation and deconjugation (Ridlon et al. 2006). Better understanding of the mechanisms involved in CDCA‐ and LCA‐induced regulation of paracellular permeability could be important in identifying future therapeutic strategies to tighten disrupted epithelial barrier, such as those seen in inflammatory diseases.

## Conflict of Interest

None declared.
